# Supplementary material for: Comparative analysis of mite genomes reveals positive selection for diet adaptation
Source: Commun Biol. 2021 Jun 3;4:668. doi: 10.1038/s42003-021-02173-3 (PMC8175442; doi:10.1038/s42003-021-02173-3)
Supplement: Supplementary file 2 — Supplementary Information [file 42003_2021_2173_MOESM2_ESM.pdf]

Supplemental Information

Title: Comparative analysis of mite genomes reveals positive selection for diet adaptation

Authors: Liu et al.

Correspondences: chende@bnu.edu.cn; weil9@mail.sysu.edu.cn;

This pdf file includes:

Supplementary Figures 1-3

Supplementary Tables 1-2

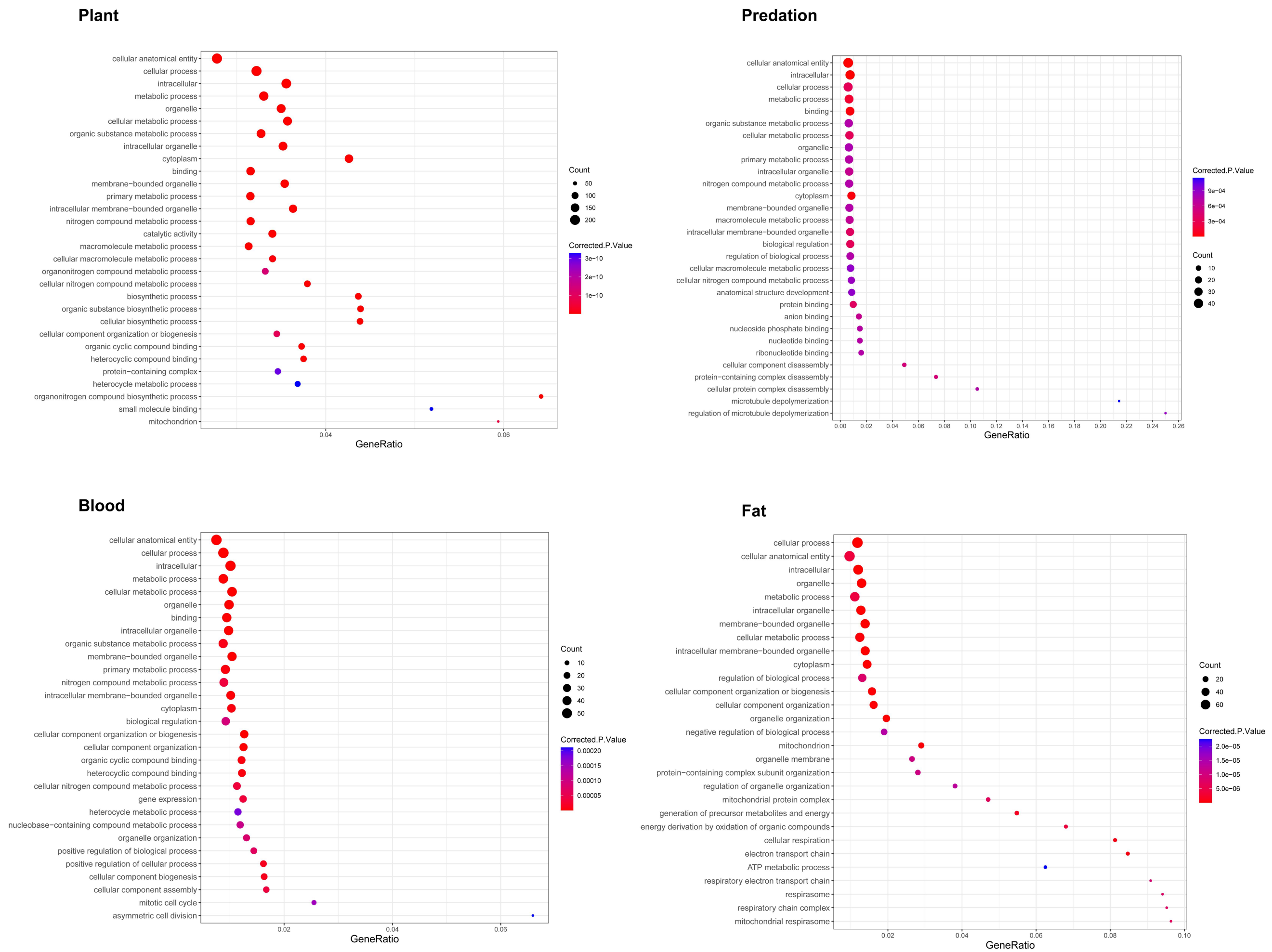

Supplementary Figure 1. Gene Ontology enrichment of PSGs. Gene Ontology enrichment of PSGs in each group generated by all the orthologous genes.

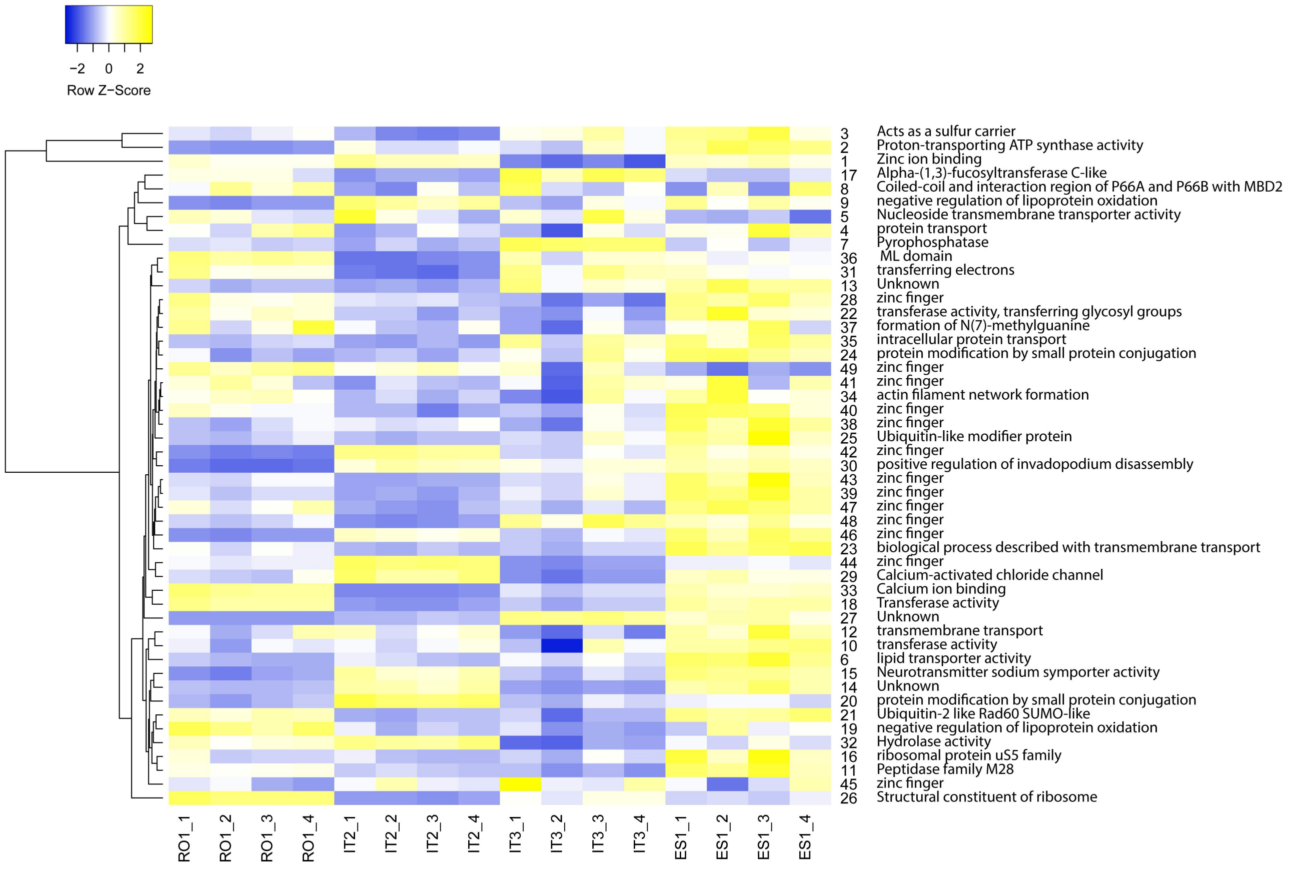

pSupplementary Figure 2. Top 50 gene expression in four population of spider mite. Heatmap and hierarchical clustering show gene expression of top 50 expressed genes (Displayed as absolute standardized RPKM values) found in four population (RO1, IT2, IT3, and ES1) of Tetranychus urticae.

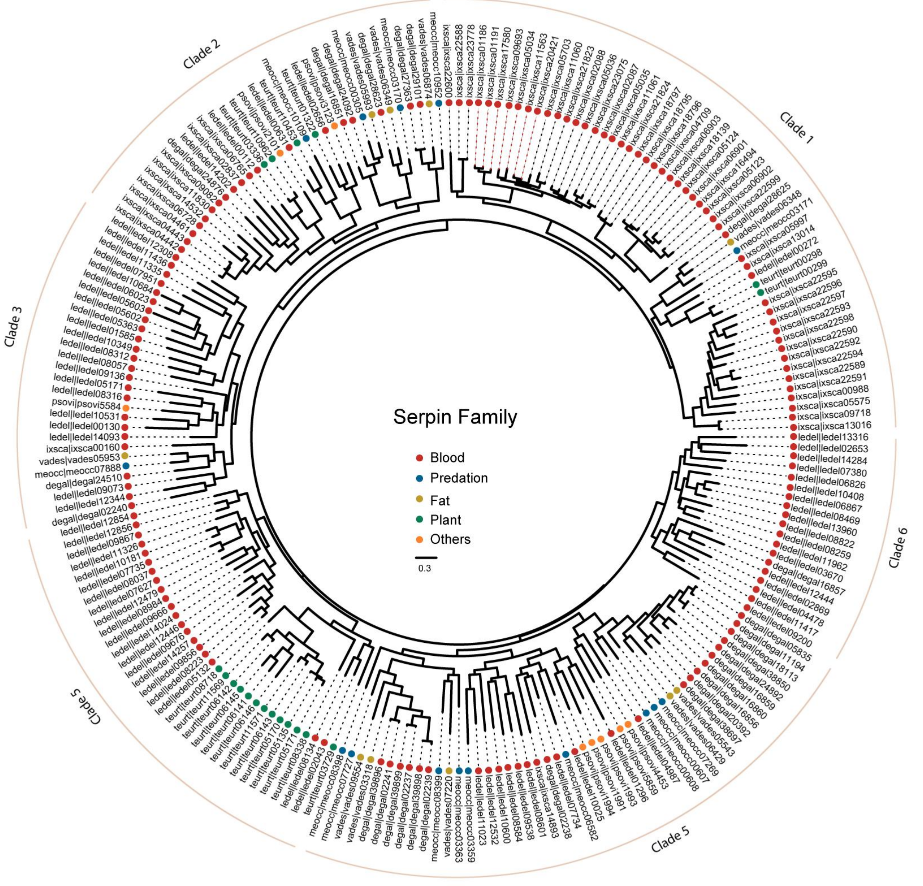

Supplementary Figure 3. Expansion of Serpin Family in blood feeding mites. A phylogenetic analysis using amino acid sequences containing the serpin domain in blood feeding group and other groups is shown. The tree was built based on Maximum Likelihood method. All nodes have significant bootstrap support based on 1000 replicates. Branches of different colors show their

taxa, as shown in the figure legend. The outer numbers indicate the six clades derived from this study, the ranges of which are marked by orange circles.

Supplementary Table 1. Completeness of genomes and genes

Latin name	BUSCO_Genome	BUSCO_Gene
Ixodes scapularis	C:95.4%[S:58.1%,D:37.3%],F:0.3%,M:4.3%,n:303	C:95.8%[S:84.2%,D:11.6%],F:0.4%,M:3.8%,n:2934
Metaseiulus occidentalis	C:96.7%[S:93.7%,D:3.0%],F:1.0%,M:2.3%,n:303	C:98.4%[S:95.6%,D:2.8%],F:0.1%,M:1.5%,n:2934
*Dermanyssus gallinae**	C:95.0%[S:78.2%,D:16.8%],F:0.3%,M:4.7%,n:303	C:78.0%[S:67.1%,D:10.9%],F:6.2%,M:15.8%,n:2934
Tropilaelaps mercedesae	C:90.4%[S:90.1%,D:0.3%],F:5.3%,M:4.3%,n:303	C:93.3%[S:91.8%,D:1.5%],F:2.9%,M:3.8%,n:2934
Varroa destructor	C:95.7%[S:94.4%,D:1.3%],F:1.7%,M:2.6%,n:303	C:98.5%[S:97.3%,D:1.2%],F:0.1%,M:1.4%,n:2934
Varroa jacobsoni	C:96.7%[S:95.4%,D:1.3%],F:1.3%,M:2.0%,n:303	C:99.1%[S:98.2%,D:0.9%],F:0.0%,M:0.9%,n:2934
*Steganacarus magnus**	C:85.5%[S:84.2%,D:1.3%],F:7.9%,M:6.6%,n:303	C:36.9%[S:36.0%,D:0.9%],F:7.8%,M:55.3%,n:2934
*Hypochthonius rufulus**	C:89.1%[S:87.1%,D:2.0%],F:5.9%,M:5.0%,n:303	C:28.8%[S:27.9%,D:0.9%],F:6.6%,M:64.6%,n:2934
Sarcoptes scabiei	C:91.1%[S:91.1%,D:0.0%],F:4.0%,M:4.9%,n:303	C:88.9%[S:88.0%,D:0.9%],F:3.3%,M:7.8%,n:2934
*Dermatophagoides farinae**	C:95.4%[S:77.6%,D:17.8%],F:0.7%,M:3.9%,n:303	C:90.6%[S:72.6%,D:18.0%],F:1.5%,M:7.9%,n:2934
*Psoroptes ovis**	C:93.7%[S:92.7%,D:1.0%],F:2.0%,M:4.3%,n:303	C:66.7%[S:65.4%,D:1.3%],F:3.3%,M:30.0%,n:2934
Leptotrombidium deliense	C:84.5%[S:83.5%,D:1.0%],F:6.3%,M:9.2%,n:303	C:84.5%[S:82.4%,D:2.1%],F:3.9%,M:11.6%,n:2934
Dinothrombium tinctorium	C:80.5%[S:41.9%,D:38.6%],F:4.3%,M:15.2%,n:303	C:87.6%[S:52.7%,D:34.9%],F:1.9%,M:10.5%,n:2934
*Brevipalpus yothersi**	C:83.9%[S:82.2%,D:1.7%],F:3.0%,M:13.1%,n:303	C:49.0%[S:47.1%,D:1.9%],F:5.8%,M:45.2%,n:2934
Tetranychus urticae	C:93.7%[S:89.4%,D:4.3%],F:1.3%,M:5.0%,n:303	C:94.2%[S:88.2%,D:6.0%],F:0.4%,M:5.4%,n:2934
Stegodyphus mimosarum	C:88.1%[S:85.1%,D:3.0%],F:5.3%,M:6.6%,n:303	C:88.2%[S:85.1%,D:3.1%],F:8.0%,M:3.8%,n:2934

Supplementary Table 2. Gene family expansion and contraction in each group

Clade	Diet group	ID	Annotation	Expansion/Contraction	Ancestor_size	Node_size	*P.value*
Dermanyssus gallinae	blood	OG0000075	TxS	Expansion	4	14	5.22E-07
Ixodes scapularis	blood	OG0000075	TxS	Expansion	4	58	1.59E-23
Leptotrombidium deliense	blood	OG0000103	CPVL	Expansion	16	30	2.97E-05
Dermanyssus gallinae	blood	OG0000103	CPVL	Expansion	7	32	4.30E-16
Ixodes scapularis	blood	OG0000103	CPVL	Expansion	8	24	2.24E-05
Leptotrombidium deliense	blood	OG0000515	MALRD1	Expansion	4	18	1.05E-08
Ixodes scapularis	blood	OG0000515	MALRD1	Expansion	2	8	0.00216866
Leptotrombidium deliense	blood	OG0000186	NA	Expansion	12	24	6.36E-05
Ixodes scapularis	blood	OG0000186	NA	Expansion	6	24	1.07E-06
Dermanyssus gallinae	blood	OG0000318	NA	Expansion	3	7	0.00265511
Ixodes scapularis	blood	OG0000318	NA	Expansion	3	10	0.00196722
Leptotrombidium deliense	blood	OG0000181	FMO5	Expansion	6	25	8.02E-11
Ixodes scapularis	blood	OG0000181	FMO5	Expansion	6	18	0.000185547
Dermanyssus gallinae	blood	OG0000059	serpin	Expansion	9	16	0.00139288
Ixodes scapularis	blood	OG0000059	serpin	Expansion	13	34	6.08E-06
Dermanyssus gallinae	blood	OG0000195	4CL	Expansion	8	15	0.000826224
Ixodes scapularis	blood	OG0000195	4CL	Expansion	10	46	3.14E-12
Leptotrombidium deliense	blood	OG0000058	TTPAL	Expansion	9	15	0.00771831
Ixodes scapularis	blood	OG0000058	TTPAL	Expansion	17	42	1.75E-06
Metaseiulus occidentalis	predation	OG0000074	TIGD6	Expansion	9	16	0.0048569
Stegodyphus mimosarum	predation	OG0000074	TIGD6	Expansion	17	40	0.0010036
Dinothrombium tinctorium	predation	OG0000318	NA	Expansion	2	10	5.86E-06
Stegodyphus mimosarum	predation	OG0000318	NA	Expansion	1	8	0.00271694

Dinothrombium tinctorium	predation	OG0000075	TxS	Expansion	6	11	0.00790888
Metaseiulus occidentalis	predation	OG0000075	TxS	Expansion	4	18	2.09E-08
Stegodyphus mimosarum	predation	OG0000075	TxS	Expansion	1	8	0.00271694
Dinothrombium tinctorium	predation	OG0000125	nas-15	Expansion	12	19	0.0064872
Stegodyphus mimosarum	predation	OG0000125	nas-15	Expansion	12	37	7.74E-05
Dinothrombium tinctorium	predation	OG0000446	Nhe2	Expansion	4	11	0.000202732
Stegodyphus mimosarum	predation	OG0000446	Nhe2	Expansion	1	9	0.00134242
Dinothrombium tinctorium	predation	OG0000080	KIAA1919	Expansion	13	33	6.89E-09
Metaseiulus occidentalis	predation	OG0000080	KIAA1919	Expansion	13	22	0.00198076
Dinothrombium tinctorium	predation	OG0000139	NA	Expansion	7	14	0.00136658
Stegodyphus mimosarum	predation	OG0000139	NA	Expansion	13	55	2.99E-08
Dinothrombium tinctorium	predation	OG0000761	ADAMTS9	Expansion	1	4	0.00348817
Stegodyphus mimosarum	predation	OG0000761	ADAMTS9	Expansion	1	9	0.00134242
Dinothrombium tinctorium	predation	OG0000462	NA	Expansion	3	7	0.00531858
Metaseiulus occidentalis	predation	OG0000462	NA	Expansion	1	4	0.00408582
Common ancestor	plant	OG0000078	NA	Expansion	13	23	9.39E-05
Common ancestor	plant	OG0000143	FASN	Expansion	7	14	2.38E-04
Common ancestor	plant	OG0000061	Cyp18a1	Expansion	11	18	1.88E-03
Common ancestor	plant	OG0000444	NA	Expansion	4	8	3.67E-03
Common ancestor	plant	OG0000176	CTSB	Expansion	8	13	5.64E-03
Common ancestor	plant	OG0000811	DNA2	Expansion	1	3	9.53E-03
Common ancestor	fat	OG0000075	TxS	Contraction	4	0	1.09E-05
Common ancestor	fat	OG0000113	H3F3A	Contraction	4	0	1.09E-05
Common ancestor	fat	OG0000148	NA	Contraction	4	0	1.09E-05
Common ancestor	fat	OG0000083	FAR1	Contraction	12	7	0.000385403

Common ancestor	fat	OG0000103	CPVL	Contraction	7	3	0.000406356
Common ancestor	fat	OG0000087	mks-3	Contraction	4	1	0.000799169
Common ancestor	fat	OG0000098	ybhF_2	Contraction	5	2	0.00164907
Common ancestor	fat	OG0000126	hta1	Contraction	11	7	0.00216877
Common ancestor	fat	OG0000371	LGMN	Contraction	2	0	0.00276625
Common ancestor	fat	OG0000074	TIGD6	Contraction	3	1	0.00721711
Common ancestor	fat	OG0000130	STOM	Contraction	3	1	0.00721711
Common ancestor	fat	OG0000059	serpin	Contraction	9	6	0.00895224
Common ancestor	fat	OG0000099	NA	Contraction	9	6	0.00895224
