# Supplementary material for: Comparative analysis of mite genomes reveals positive selection for diet adaptation
Source: Commun Biol. 2021 Jun 3;4:668. doi: 10.1038/s42003-021-02173-3 (PMC8175442; doi:10.1038/s42003-021-02173-3)
Supplement: Supplementary file 3 — Description of Supplementary Files [file 42003_2021_2173_MOESM3_ESM.pdf]

Description of Additional Supplementary Files

File name: Supplementary Data 1

Description: Pseudogene gene list.

File name: Supplementary Data 2

Description: Raw data of Figure2,3,5,6, Supplementary figure 1.

File name: Supplementary Data 3

Description: Top 200 highly expressed gene list in herbivorous species.
